# Charity Mission: Bringing Meaningful Impact and Sustainability

**DOI:** 10.1055/s-0043-1772588

**Published:** 2023-10-05

**Authors:** Joon Pio Hong, Editor-in-Chief Archives of Plastic Surgery

**Affiliations:** 1 Department of Plastic Surgery, Asan Medical Center University of Ulsan, Seoul, Korea

**Figure:**
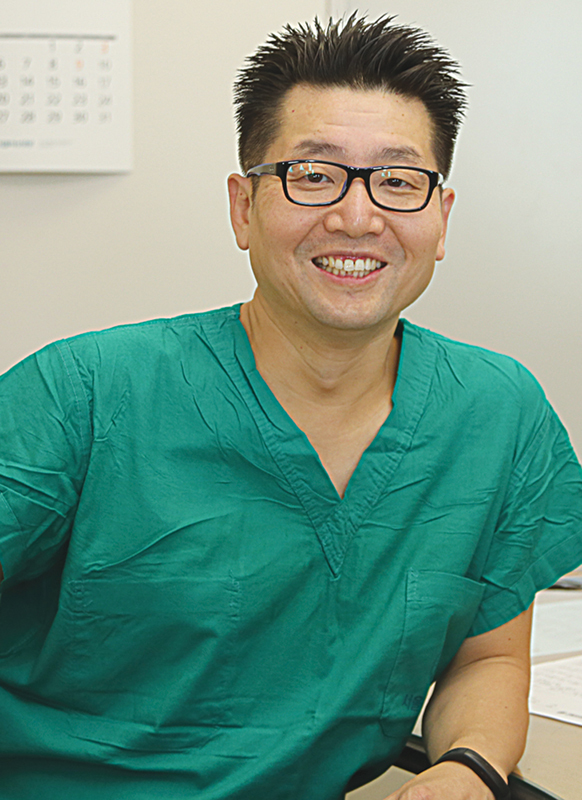
Joon P. Hong, MD, PhD, MMM

As plastic surgeons, we are privileged to answer a helping call to many patients that are in need. These patients arrange from congenital anomalies, burns, various soft and bone tissue defects after cancer and trauma to providing solutions to what was once thought difficult such as allotransplant, transgender surgery, and even complications arising from the abnormality of lymphatic functions. As plastic surgeons we have achieved remarkable advancement in the field of aesthetic and reconstructive surgery. However, there are enormous disparity between different parts of the world where reconstruction may not even be available due to lack of plastic surgeons or resources. We hear stories about how people unnecessarily amputate their limbs, form disabilities from poor care, and live with congenital deformities that may leave a stigma in their society. As plastic surgeons we could bring lasting impact to these countries by sharing knowledge, providing care, and sending resources. We could help them dream what can be possible and help them a step closer to realizing this dream. We have many wonderful charities and missions in the field of plastic surgery that has continuously served these underprivileged patients around the world. We have many surgeons who work with charity missions or even on their own provide help. But the progress remains slow despite the current work by various institutions and individuals. So, what do we need to do to reach more patients and to help them transform their lives?

There are several factors involved in surgical missions. But the key to successful mission is to carefully plan based on surgical needs, work with local partners to coordinate the mission, and help them acquire the necessary skills plus resources to manage these patients, and to be responsible in practicing good ethics.

I have taken part in many missions and although all missions are different in need, stable mission that does not address the imminent crisis of war or conflict gives you the opportunity to prepare accordingly maximizing the efficiency of the mission. Often local partners will triage and schedule cases, which allows to efficiently prepare the resources that are needed as well as to designate the proper surgeon with good experience for the required task. Having similar patient groups will allow a better learning experience for the local partners to enhance their knowledge and skills as well as postoperative care. The best way to reach all the patients in need is to train the local partners as they will go out in the field to serve the patients and will train more surgeons ultimately increasing the care providers. Only teaching them how to fish will allow them to build the right capacity to address the problems and empower them to transform the lives of their own patients. Keep in touch with the local partners answering their questions and helping them to find solutions even after leaving the mission. Finally, often overlooked, is the issue of ethics. As surgeons, we practice patient safety as a habit. The same must be applied in any missions despite the limited resources they might have. If you are going to let trainees operate, you but be there to guide them in every step of the surgery. Educating the patient and family and obtaining informed consent needs to be a routine. The best practice should be performed as same as back home. Although customs and culture can be different, we must practice to respect to our local colleagues, partners, and patients accommodating their beliefs, which will in turn lead to building trust.

During the mission, despite the tireless efforts, we can only reach a limited number of patients. We often leave with guilt not being able to help more. But knowing we have trained and educated the partners, we can leave with the notion of plating a seed. A seed that will continue to grow long after we leave. A seed that will have strong roots as we continue to help the local partners sustain the practice they learned. We should continue to be part of their journey of transformation and provide support in ways we can. For those who will embark in the journey of giving back, planning, supporting the local partners, and practicing good ethics will be the key to helping them grow and leaving a lasting and meaningful impact that will lead to a cycle of positivity and sustainability.

